# Genetic and Genomic Resources for Soybean Breeding Research

**DOI:** 10.3390/plants11091181

**Published:** 2022-04-27

**Authors:** Jakob Petereit, Jacob I. Marsh, Philipp E. Bayer, Monica F. Danilevicz, William J. W. Thomas, Jacqueline Batley, David Edwards

**Affiliations:** School of Biological Sciences, The University of Western Australia, Perth, WA 6009, Australia; jakob.petereit@uwa.edu.au (J.P.); jacob.marsh@research.uwa.edu.au (J.I.M.); philipp.bayer@uwa.edu.au (P.E.B.); monica.danilevicz@research.uwa.edu.au (M.F.D.); william.thomas@research.uwa.edu.au (W.J.W.T.); jacqueline.batley@uwa.edu.au (J.B.)

**Keywords:** soybean, germplasm, genomics, assemblies, pangenome, genetics, breeding, genetic variation, databases

## Abstract

Soybean (*Glycine max*) is a legume species of significant economic and nutritional value. The yield of soybean continues to increase with the breeding of improved varieties, and this is likely to continue with the application of advanced genetic and genomic approaches for breeding. Genome technologies continue to advance rapidly, with an increasing number of high-quality genome assemblies becoming available. With accumulating data from marker arrays and whole-genome resequencing, studying variations between individuals and populations is becoming increasingly accessible. Furthermore, the recent development of soybean pangenomes has highlighted the significant structural variation between individuals, together with knowledge of what has been selected for or lost during domestication and breeding, information that can be applied for the breeding of improved cultivars. Because of this, resources such as genome assemblies, SNP datasets, pangenomes and associated databases are becoming increasingly important for research underlying soybean crop improvement.

## 1. Introduction

Cultivated soybean (*Glycine max*) is a major protein and oil crop and reached a worldwide production of 349 million tons in 2018, equivalent to a total export value of USD 59 billion (http://www.fao.org/faostat, accessed on 15 October 2021).

*G. max* is a palaeopolyploid (2n = 20) that has undergone multiple genome duplication und subsequent re-diploidisation events with simultaneous rearrangements among chromosomes [1,2,3], which resulted in up to 12 occurrences of a given genome region in *G. max* [3,4].

The global importance of soybean as a crop enabled the growing amount of soybean breeding research on varieties ranging from wild and semi-wild relatives to domesticated landraces and modern elites, including genome and transcriptome sequencing, functional assays, phenotype and trait discovery. The wide range of assemblies, pangenome and variant resources as well as databases support researchers in studying soybean.

The progress of soybean research resources in the last decade has been recently reviewed with a focus on gene discovery [5]. We expand this by summarizing resources that support researchers in the field of soybean breeding research.

**Figure 1:**
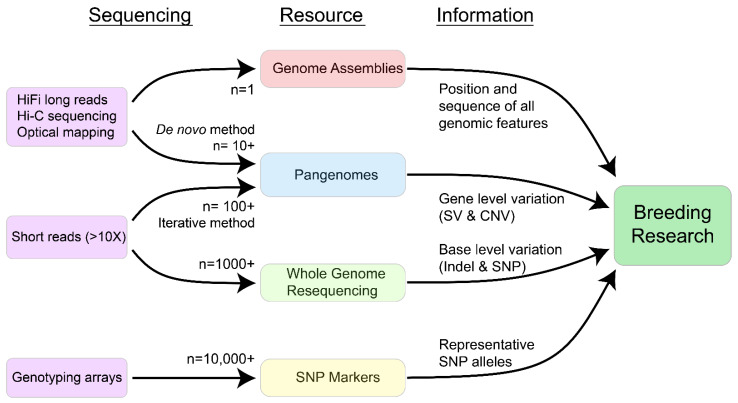
Sequencing required and information gained from core genomic resources for soybean breeding research (n—number of individuals included in a typical study).

In this review, we elucidate milestones in soybean genetics and genomics research (Figure 1) and provide details on the currently available soybean genetic and genomic databases. We detail available marker technologies for soybean and summarize soybean whole-genome resequencing studies as gold standard for variation studies across populations. We provide the step-by-step development of the current high quality reference genomes and pangenomes and highlight the challenges of data interoperability, metadata annotation and scarcity of associated data, including data for proteomics, metabolomics and phenomics, which limit the application of these data for crop improvement. Finally, we propose approaches that may support more integrated data management and analysis; so, as databases continue to improve and expand, they can be applied for the improvement of this important crop.

## 2. Main

### 2.1. SNP Marker Arrays

SNP marker arrays are a cost-effective option for capturing genetic variation across a population. These marker arrays report the allelic state of specific loci for individuals across the genome, designed to provide an overview of the genome, or target regions of interest, with applications both in breeding and research [6]. The first major genotyping array spanning the soybean genome, the Soy50KSNP array [7], allowed researchers to characterize 52,041 variant sites. This was applied to genotype the 18,480 domesticated and 1168 wild accessions in the USDA Soybean Germplasm Collection [8]. Genotyping using this array can be carried out in conjunction with trait association analysis, such as GWAS, to identify regions underlying agronomically important traits related to seed composition [9,10,11], flooding tolerance [12,13,14] and sudden death syndrome [15,16]. Denser marker genotyping arrays were subsequently developed, including the 180K AXIOM^®^ SoyaSNP array [17] and NJAU 355K SoySNP array [18], allowing for more in depth inquiry into the landscape of genomic diversity in soybean and providing insights into the history of soybean domestication [19,20,21].

Although dense SNP arrays are preferred when studying soybean evolution, studies suggest that, to obtain maximum efficiency in genomic prediction breeding models, only 1000–2000 markers are required [22,23]. As a result, there have been advancements towards targeting smaller, non-redundant sets of informative markers. The BARCSoySNP6K was developed for cost-effective recombination tracing in biparental populations [24,25], though it has also found utility in global population research [26,27].

Recent progress in bioinformatics has maximized the lower density genotype information gained from SNP marker arrays and genotyping-by-sequencing (GBS) through imputation using haplotypes identified in more detailed whole-genome resequencing (WGRS) data [28,29]. The GmHapMap was constructed using 1007 whole-genome resequenced individuals and enables the inference of allelic states with 96% accuracy at all SNP positions across the genome from only the 42,508 SNPs genotyped by the Soy50KSNP array [30].

### 2.2. Whole-Genome Resequencing

Currently, the gold standard method used to map genetic diversity in detail for breeding and genomic research is Whole-Genome Resequencing (WGRS) [31]. WGRS involves low-coverage sequencing individuals with short reads before being aligned to a genomic reference to identify nucleotides or regions that vary from the reference. Compared to SNP marker arrays, WGRS is often more expensive per individual, though it can provide high-density genome-wide allelic information for all loci in a reference [32]. Beyond small variants, such as SNPs, resequencing individuals can allow for the identification of structural diversity, such as copy number variation underlying soybean cyst nematode resistance [33].

The first major population-level WGRS project for soybean was published in 2010 for 17 wild and 14 domesticated soybean individuals, sequenced to an average depth of 5X [34]. This dataset was used in one of the first whole-genome investigations of structural variation, which revealed low levels of linkage disequilibrium decay compared to other plant species [34]. The same dataset was later used to identify a gene underpinning salt tolerance in wild soybean [35].

In 2015, WGRS increased substantially with the release of data for 302 wild and domesticated individuals for GWAS, characterizing selective signals related to domestication and improvement [36], as well as maternal lineages in the chloroplast genome [37]. Since 2015, the global soybean community steadily accumulated WGRS data through a series of projects, with an increasing focus on characterizing regional germplasm collections in Brazil [38], China [39,40], Canada [41], the USA [42], Japan [43] and Korea [29]. The largest soybean WGRS dataset to date contains 2898 wild and domesticated accessions, the majority originating from China, which was aligned to a Zhonghuang 13-based graph pangenome [44].

The growing availability of larger, more diverse WGRS datasets, including previously unstudied exotic individuals, holds tremendous promise for integrated research studying the underlying genomic basis of trait variability in soybean lineages.

High-quality genome assemblies for domesticated and wild soybean support researchers and breeders improving and adapting soybean for changing climate conditions, associated biotic and abiotic stresses, or market changes in soybean demand. Current, assemblies for domesticated soybean accessions capture the genetic diversity from the USA and China, but there is no high-quality assembly for the Brazilian germplasm. Genome assemblies for *G. max* are complemented by wild and perennial *Glycine* assemblies that allow researchers to identify changes in modern soybean due to domestication, as well as potentially beneficial genetic diversity that may have been lost.

### 2.3. Genome Assemblies

**Figure 2:**
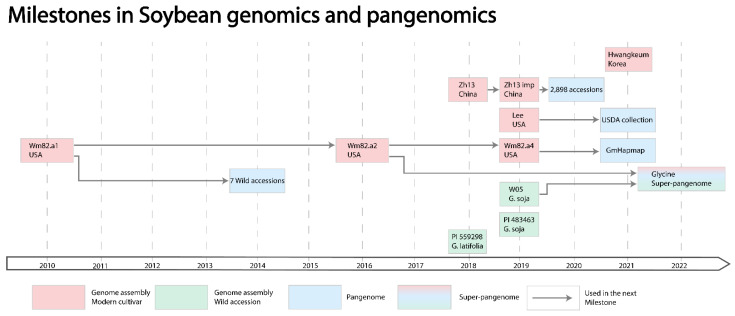
Milestones progressing soybean genomics and pangenomics. Red boxes indicate genome assemblies of modern cultivars (Wm82.a1/a2/a3—*G. max* Williams82, first, second and fourth revision, Lee—*G. max* Lee, Zh13, Zh13 imp—*G. max* Zhonhuang 13 and Zhonghuang 13 improved), green boxes indicate wild genome assemblies (W05—*G. soja* accession W05, PI 483463—*G. soja* accession, PI 559298—*G. latifolia* accession), blue boxes indicate pangenomes, including the used accessions for their construction, and the red-blue-green box depicts the *Glycine* super-pangenome, including *G. max*, *G. soja* and 7 perennial *Glycines*. Arrows indicate the use of a constructed genome assembly in a later study.

The first assembly for cultivated soybean, *Glycine max* var. Williams 82 (Wm82.a1), was published in 2010 [3] (Figure 2), with a size of 950 Mb and 46,430 gene models [3,45], which is more than eight times the size of the *Arabidopsis thaliana* genome and twice the size of many other legumes [46]. The assembly identified multiple rounds of *Glycine*-specific genome duplication that has led to 75% of genes becoming non-unique, and partially explains the large, repetitive *G. max* genome [3]. This assembly provided a foundation for functional genomics in soybean to accelerate crop trait dissection and support breeding programs. The Wm82.a1 assembly was ordered based on linkage maps using a limited number of markers and recombinant inbred lines, resulting in limited assembly quality in regions with low marker density [47]. In 2016, a new version of the *G. max* Wm82 assembly, Wm82.a2, which was published using two high-density linkage maps with a total assembly size of 978.5 Mb [47] (Figure 2). In 2019, the Wm82 assembly was further improved (Wm82.a4), closing 3600 gaps and adding another 5 Mb to the assembly size [48] (Figure 2). The same study also released an assembly for the southern US accession Lee, with an assembly size of 985 Mb and a high structural similarity when compared with Wm82.a4 (Figure 2). Both the Wm82.a4 and Lee assemblies represent much of the genetic diversity present in USA soybean cultivars, building a strong foundation for US soybean genetic research.

Outside the USA, a reference genome for the Chinese soybean cultivar Zhonghuang 13 was released in 2018. The assembly size was 1025 Mb with 52,021 gene models and 250,000 structural variations compared to Wm82.a2 [49] (Figure 2). This assembly was later improved using PacBio reads, optical mapping and Hi-C sequencing, and the total number of protein coding genes increased to 55,443 by integrating RNAseq data into the annotation [50] (Figure 2).

Following the draft assemblies of seven wild soybean accessions in 2014 [51], the first reference-grade assemblies of two wild soybean accession were published in 2019 (Figure 2). The *G. soja* accession W05 was assembled with a size of 1013 Mb and 55,539 genes [52], identified an inversion in the seed color locus, a translocation between chromosome 11 and 13, and highlighted copy number variations for several gene clusters [52] (Figure 2). A second *G. soja* accession PI 483463 was also sequenced, with a 962 Mb assembly, demonstrating significant sequence diversity [48]. An assembly for *Glycine latifolia* accession PI 559298, a perennial relative, was released in 2018 [53] presenting high levels of genetic diversity and agronomically favorable traits, including sclerotinia stem rot and soybean rust resistance that are absent in *G. max* [54,55,56,57]. The assembly of 939 Mb and 54,475 genes included hundreds of candidate disease-resistance genes, including 367 LRR genes, less than the 467 LRR genes found in *G. max* [36,53].

Recently, a genome assembly of the popular Korean soybean cultivar Hwangkeum, known for its resistance to all the USA soybean mosaic virus strains, was released with an assembly size of 933.12 Mb and 58,550 genes [58] (Figure 2). While SNPs, indels and structural variants were identified when comparing Hwangkeum with Wm82.a4, no large genomic rearrangements were identified, which is in contrast to four large scale chromosomal rearrangements identified between Wm82.a4 and Zhonghuang 13 [49,50].

The global importance of soybean as a crop is reflected in the regularity of improvements to soybean genome assemblies. The reference assembly for Wm82 has been improved twice since its initial release in 2010, and together with the reference assemblies for Lee, Zhonghuang 13 and Wm82, the latter of which has been improved twice since its initial release in 2010, provide the foundation of modern soybean research.

### 2.4. Pangenomes

Comparative genomic studies have demonstrated that single reference genome assemblies do not represent the full genomic diversity of a species. To address this, pangenomes have been assembled that represent the gene content of a species rather than of a single individual [59,60,61]. Pangenomes have been assembled for several plant species, such as banana [62], sorghum [63], bread wheat [64], *Brassica oleracea* [60], *Brassica napus* [65], the *Brassica* genus [66], chickpea [67], tomato [68], sunflower [69], pigeon pea [70], cotton [71] and rice [72]. These studies have revealed extensive gene presence/absence variation and that some genes that are not present in all accessions may have important biological functions, such as biotic and abiotic stress tolerance.

The first soybean pangenome was published in 2014 and was one the first pangenomes developed in plants [51] (Figure 2). The study mapped whole-genome resequencing data for seven representative *G. soja* accessions to the Wm82.a1 reference and identified 3.63 to 4.72 million SNPs, 0.5 to 0.77 million indels and a total of 338 genes that were absent in the *G. max* reference. Variable genes were enriched for defense response, cell growth and photosynthesis [51].

Soybean pangenomics expanded in 2020 with the analysis of 2898 accessions, including the *de novo* assembly of 26 individuals representing distinct diversity clusters [44] (Figure 2). These 26 accessions were combined into a graph-based pangenome using vg [73] with Zhonghuang 13 as the primary reference genome. Finally, data from the full set of 2898 accessions were mapped to the pangenome graph and structural variants identified. This process identified a total of 57,492 gene families, of which only 35.9% were present in all 27 accessions [44]. Variable gene families were more diverse and had a higher rate of positive selection compared to core genes, and they were also enriched for abiotic and biotic stress response annotation. The study identified 14.6 million SNPs and 12.7 million indels when comparing the pangenome with the Zhonghuang 13 reference [44]. The wealth of small and variant information collated in this dataset has been used to characterize structural variants associated with iron use efficiency and flowering time as well as inversions and gene fusion events associated with soybean domestication [44].

Two further pangenome studies were published in 2021 [74,75] (Figure 2). PanSoy was constructed using the GmHapMap dataset [30], processed with the EUPAN pipeline [76] based on the Wm82 reference, and resulted in a total pangenome size of 1086 Mb with 54,531 genes, including 1659 novel genes. Of these genes, 7% were variable and enriched for annotations associated with the regulation of immune and defense responses, signaling and plant development [74]. The other pangenome was constructed using a previously published iterative method [60] and based on the Lee soybean assembly. It represents the USDA soybean collection, including wild lines, landraces and modern cultivars. The resulting pangenome had an assembly size of 1213 Mb with 51,414 genes [75]. Of these, 13.2% were variable and enriched in annotations associated with response to biotic and abiotic stress, including defense response, response to abscisic acid and response to salt stress. In addition, the USDA soybean pangenome identified genes that changed in frequency when comparing individuals with different breeding histories [75]. These three pangenomes capture the majority of the genomic diversity present in *G. max* and *G. soja*. However, the overall genetic diversity in this gene pool still remains low and limits the crops’ potential in yield and resilience [77].

The expansion of the known gene pool in soybean is the focus of the most recent study by Zhuang, et al. [78] (Figure 2), which *de novo* assembled five diploid perennial Australian *Glycine* species (2n = 40), *G. falcata*, *G. stenophita*, *G. cyrtoloba*, *G. syndetika* and *G. tomentella* D3 and the perennial Australian allopolyploid *G. dolichocarpa* (2n = 80) at the chromosome level. The assembly sizes of the 5 diploids range from 941 to 1374 Mb with 55,376 to 58,312 protein coding genes and the allopolyploid *G. dolichocarpa* had an assembly size of 1948 Mb and 113,697 genes. The assembled diploid perennial genomes and 26 selected annual soybean genomes were then used to construct a super-pangenome framework that annotated 109,827 genes in the pool of perennials with 29% perennial core genes and 129,006 genes in the annuals with 24.5% annual core genes. Of the perennial core genes, 56.2% overlapped with annual core genes, 27.2% with variable annual genes and 16.6% were perennial specific. A total of 82.3% of variable perennial genes were not found in the annual gene pool. The identification of perennial specific genes is the first step to expand soybean pangenomics across species boundaries and links genetic variation with phenotypes of agronomic importance.

### 2.5. Databases and Tools for Explorative Data Analysis

With the growing quantity and diversity of genetic and genomic information for soybean, there is a requirement for the integration of data to improve gene annotation and to discover associations between allelic variants and agronomic traits. There are currently several relevant soybean datasets. For example, SoyKB [79] and SoyBase [80] offer curated genomic and genetic datasets, including epigenetic maps, gene expression data, regulatory RNA data, genomic sequence variants and pangenome gene visualization. These databases are continuously updated to host soybean genome analysis results [74] and are employed by the community for biological analyses, including Gene Ontology enrichment [74], QTL mapping and gene identification [81], quantitative disease resistance estimation [82] and the identification of homologous genomic features in related species [83]. A list of online soybean databases is given in Table 1. Across the different databases, users can find tools to explore and visualize genetic maps, soybean mutant lines, gene families and characterize differential gene expression.

The value of genetic and genomic data is limited without associated phenotypic data. Phenotypic data have allowed breeders to identify QTLs and SVs associated with soybean yield and performance under abiotic stresses [111]. Several phenotypic datasets are hosted in the databases described in Table 1. The use of information-dense phenotype datasets can improve the association of genetic markers with crop traits [112]. For example, a multi-environment trial using 393 individuals from the SoyNAM (www.soynam.org, accessed on 15 October 2021) population used high throughput drone images to estimate above ground biomass. The derived phenotype data were used to identify genetic loci associated with biomass production at different times during crop growth [113]. Another study used image data from 5555 soybean SoyNAM lines in a GWAS, uncovering QTLs on chromosome 19 associated with average canopy coverage and increased yield [110]. With the expansion of genetic and genomic datasets, combined with high throughput phenotypic analysis, we can expect to gain a greater understanding of how genomic diversity in this crop species underpins trait diversity, information that is valuable for applied crop improvement.

The increase in genomic and phenotypic datasets for soybean and the diversity of databases provides a challenge for integrative soybean analysis as datasets are often scattered across multiple repositories, making it hard for researchers to find all the relevant information that could be used for analysis. Although SoyBase and SoyKB offer central hubs to retrieve genotypic and genetic information across multiple varieties, other ‘omics’ datasets (e.g., proteomics, metabolomics and phenomics) are not so easily found. Many published datasets have relatively poor metadata, limiting detailed analysis. The Planteome and other plant ontology references serve as standards to assist semantic integration among different datasets [114,115]. For plant phenotyping, the MIAPPE guidelines have forms suggesting the minimal information that is necessary to describe in the metadata to enable other researchers to benefit from the data [116]. Adhering to data sharing guidelines and structures will enable researchers to explore previously published data more effectively, and leverage soybean genetic diversity for crop improvement.

## 3. Conclusions and Future Perspectives in Breeding

Finding novel sources for environmental adaptation is fundamental to support breeding approaches. Genome-environment association (GEA) in conjunction with GWAS have been used to predict drought [117,118] and heat tolerance [119,120] in closely related legumes, such as the common bean, which has been proposed as a diploid model for soybean [121]. Enabled by the availability of a wealth soybean marker datasets, GEA will also be an excellent option to study soybean environment adaptation in the future. Furthermore, the availability of genomic datasets and connected phenotypic and marker databases also builds a foundation for next-generation breeding technologies, such as genomic prediction [122], genome-wide scans of selection signatures [123], machine learning [124] and speed breeding [125]. The high-quality datasets available for soybean also enable the use of genomic-assisted backcrossing and replace marker-assisted backcrossing, which will accelerate future soybean breeding.

New technologies, such as long-read sequencing, have been used to generate modern high-quality reference genomes and to *de novo* assembly of more than 20 accessions in pangenomes. We believe that long-read sequencing is poised to replace WGRS as the gold standard for high-fidelity variation mapping across populations, with the construction of larger and larger *de novo* assembled pangenomes. Pangenomes are on the verge of expanding into the higher level taxon, which has been demonstrated by Zhuang, Wang, Li, Hu, Fan, Landis, Cannon, Grimwood, Schmutz, Jackson, Doyle, Zhang, Zhang and Ma [78], and will soon start to address questions in functional genomics to enable super-pangenomics-guided breeding.

With a wealth of published soybean (pan-)genomes, genomics has firmly established itself as one of the basic tools of soybean plant breeders’ toolkit. In this review, we gave an overview of the available data and germplasm resources for soybean researchers and breeders. The valuable data stored within these resources enables new approaches to breed soybean cultivars to meet the challenges posed by a growing world population in a warming climate.

## Figures and Tables

**Table 1:** Available database resources for soybean genome investigation and computational tools.

Data Type	Database	Description	Website	
Genome and genetic data	SFGD—Soybean Functional Genomics Database	Integration-friendly genome, transcriptome and protein data for the functional characterization of soybean pathways. Has a dataset, focused on soybean acyl–lipid pathways	http://bioinformatics.cau.edu.cn/SFGD/, accessed on 15 October 2021	[84]
	Plant-Impute DB	Database for genotype imputation using high-quality reference panels	https://gong_lab.hzau.edu.cn/Plant_imputeDB/, accessed on 15 October 2021	[85]
	GmHapMap—Haplotype Map	Haplotype map constructed using genome sequence data from 1007 soybean accessions, with 4.3 M SNPs identified	https://soybase.org/projects/SoyBase.C2020.01.php, accessed on 15 October 2021	[30]
	SoyKB—Soybean knowledge base	Soybean data hub with genomic and genetic information linked to external datasets	https://soykb.org/, accessed on 15 October 2021	[79]
	SGMD—Soybean genomics and microarray database	Integrated view of the interaction of soybean with the soybean cyst nematode and contains genomic, EST and microarray data with embedded analytical tools, allowing the correlation of soybean ESTs with their gene expression profiles	https://www.hsls.pitt.edu/obrc/index.php?page=URL1096997457, accessed on 15 October 2021	[86]
	SoyTEdb—Soybean Transposable Element database	Database of transposable elements identified by genetic and physical maps based on the Glyma1.01 assembly	https://www.soybase.org/soytedb/, accessed on 15 October 2021	[87]
	DAIZUbase	Genome visualization and data mining tools (Gbrownse, Unifiedmap, Geneviewer and BLAST)	https://daizubase.daizu.dna.affrc.go.jp/, accessed on 15 October 2021	[88]
	LegumeIP V3	Translational genomics, offering tools to analyze gene expression data and pathway analysis	https://www.zhaolab.org/LegumeIP/gdp/, accessed on 15 October 2021	[89]
	SoyBase	Integrates genetic and genomic data, including QTLs and GWAS for several hybrid lines. Facilitates BLAST using soybean pangenome of all cultivars within the database	https://soybase.org/soyseq/, accessed on 15 October 2021	[80,90]
	Legume Federation	Visualization of genotype comparison, genome context viewer, gene annotation and visualiz+ation, synteny, QTLS and genetic markers search. SNPs and GWAS results available	https://www.legumefederation.org/en/tools/, accessed on 15 October 2021	
	Phytozome	Up-to-date repository of genome assemblies and annotation. Useful BLAST functionality between species	http://www.phytozome.net/soybean, accessed on 15 October 2021	[91]
	PlantGSAD v2	Numerous gene set annotations, including metabolic pathways and customized SEA annotations and integrated visualization features	http://systemsbiology.cau.edu.cn/PlantGSEAv2/, accessed on 15 October 2021	[92]
	PlantGDB	Soybean genome and annotation tools for comparative genomics	https://www.plantgdb.org/GmGDB/, accessed on 15 October 2021	[93]
	SoyTB—Transporter	Comparative analysis of transporter genes in 47 plant genomes and transcriptomes	http://artemis.cyverse.org/soykb_dev/SoyTD/, accessed on 15 October 2021	[94]
	PCMDB—Plant cell markers database	Cell markers from 6 plant species to label 263 cell types across 22 tissues	www.tobaccodb.org/pcmdb/, accessed on 15 October 2021	[95]
	SoyVCF 2 Genomes	Compares the user-supplied genomic data in the database for the identification of the closest soybean relative in a 222 germplasm collection	http://pgl.gnu.ac.kr/soy_vcf2genome/, accessed on 15 October 2021	[96]
	SNPViz v2.0	Web-based tool for the visualization of large-scale haplotype blocks with detailed SNPs and indels grouped by their chromosomal coordinates, along with their overlapping gene models, phenotype to genotype accuracies, Gene Ontology (GO) annotations, protein families (Pfam) annotations, genomic variant annotations and their functional effects	http://soykb.org/SNPViz2/, accessed on 15 October 2021	[97]
Functional networks, and co-expression data	SoyFN—Soybean Functional Networks	Gene and miRNA interaction database built into functional networks, with KEGG pathways and Gene Ontology annotations	https://nclab.hit.edu.cn/SoyFN, accessed on 15 October 2021	[98]
	SoyNet	Searchable network of soybean genes for network-based functional predictions	https://www.inetbio.org/soynet/, accessed on 15 October 2021	[99]
	SoyCSN—context-specific network	Computational pipeline to analyze, annotate, retrieve and visualize context-specific network at the transcriptome and interactome levels—based on the Soybean Gene Atlas project	http://soykb.org/SoyCSN, accessed on 15 October 2021	[100]
	PlaNet	Platform of web-based tools for the visualization of whole-genome co-expression networks in multiple species, including soybean	http://aranet.mpimp-golm.mpg.de/, accessed on 15 October 2021	[101]
Protein-related data	SoyProDB—Soybean Seed Protein Database	Identification of soybean seed proteins from 2D-PAGE gels	http://bioinformatics.towson.edu/Soybean_Seed_Proteins_2D_Gel_DB/Home.aspx, accessed on 15 October 2021	[102]
	PlantEAR	Database of EAR motif-containing proteins across 71 species	http://structuralbiology.cau.edu.cn/plantEAR/, accessed on 15 October 2021	[103]
Expressed sequence tag (EST)	HarvEST	EST data filtered using the soybean genome assembly Glyma1	https://harvest.ucr.edu/, accessed on 15 October 2021	[104]
	OcsESTdb—oil crop seed EST database	EST libraries of four oilseed species with annotated sequences	http://ocri-genomics.org/ocsESTdb/, accessed on 15 October 2021	[105]
	Soybean Marker database	Linkage map of soybean genome and genetic markers	http://marker.kazusa.or.jp/Soybean/, accessed on 15 October 2021	[106]
	Rsoy—Riken Soybean	cDNA sequences for the functional analysis of genomic features	http://spectra.psc.riken.jp/menta.cgi/rsoy/index, accessed on 15 October 2021	[107]
Manual and image phenotype	RhizoVision Crown	Crown root images and phenotypic measurements of 187 soybean lines. Additionally, it offers a tool for root phenotyping	https://zenodo.org/record/5121845#.YYzkYJvmiV4, accessed on 15 October 2021	[108,109]
	SoyNAM	Phenotype and genotype data from 5555 SoyNAM lines, available through the R package NAM	https://CRAN.R-project.org/package=NAM, accessed on 15 October 2021	[110]

## Data Availability

Not applicable.

## References

[1] Doyle J.J., Egan A.N. (2010). Dating the origins of polyploidy events. New Phytol..

[2] Pfeil B., Schlueter J., Shoemaker R., Doyle J. (2005). Placing paleopolyploidy in relation to taxon divergence: A phylogenetic analysis in legumes using 39 gene families. Syst. Biol..

[3] Schmutz J., Cannon S.B., Schlueter J., Ma J., Mitros T., Nelson W., Hyten D.L., Song Q., Thelen J.J., Cheng J. (2010). Genome sequence of the palaeopolyploid soybean. Nature.

[4] Cannon S.B., Shoemaker R.C. (2012). Evolutionary and comparative analyses of the soybean genome. Breed. Sci..

[5] Zhang M., Liu S., Wang Z., Yuan Y., Zhang Z., Liang Q., Yang X., Duan Z., Liu Y., Kong F. (2022). Progress in soybean functional genomics over the past decade. Plant Biotechnol. J..

[6] Syvänen A.C. (2005). Toward genome-wide SNP genotyping. Nat. Genet..

[7] Song Q., Hyten D.L., Jia G., Quigley C.V., Fickus E.W., Nelson R.L., Cregan P.B. (2013). Development and Evaluation of SoySNP50K, a High-Density Genotyping Array for Soybean. PLoS ONE.

[8] Song Q., Hyten D.L., Jia G., Quigley C.V., Fickus E.W., Nelson R.L., Cregan P.B. (2015). Fingerprinting Soybean Germplasm and Its Utility in Genomic Research. G3 Genes|Genomes|Genetics.

[9] Leamy L.J., Zhang H., Li C., Chen C.Y., Song B.-H. (2017). A genome-wide association study of seed composition traits in wild soybean (Glycine soja). BMC Genom..

[10] Bandillo N., Jarquin D., Song Q., Nelson R., Cregan P., Specht J., Lorenz A. (2015). A Population Structure and Genome-Wide Association Analysis on the USDA Soybean Germplasm Collection. Plant Genome.

[11] Hwang E.-Y., Song Q., Jia G., Specht J.E., Hyten D.L., Costa J., Cregan P.B. (2014). A genome-wide association study of seed protein and oil content in soybean. BMC Genom..

[12] Patil G., Do T., Vuong T.D., Valliyodan B., Lee J.-D., Chaudhary J., Shannon J.G., Nguyen H.T. (2016). Genomic-assisted haplotype analysis and the development of high-throughput SNP markers for salinity tolerance in soybean. Sci. Rep..

[13] Sharmin R.A., Karikari B., Chang F., Al Amin G.M., Bhuiyan M.R., Hina A., Lv W., Chunting Z., Begum N., Zhao T. (2021). Genome-wide association study uncovers major genetic loci associated with seed flooding tolerance in soybean. BMC Plant Biol..

[14] Wu C., Mozzoni L.A., Moseley D., Hummer W., Ye H., Chen P., Shannon G., Nguyen H. (2019). Genome-wide association mapping of flooding tolerance in soybean. Mol. Breed..

[15] Wen Z., Tan R., Yuan J., Bales C., Du W., Zhang S., Chilvers M.I., Schmidt C., Song Q., Cregan P.B. (2014). Genome-wide association mapping of quantitative resistance to sudden death syndrome in soybean. BMC Genom..

[16] Zhang J., Singh A., Mueller D.S., Singh A.K. (2015). Genome-wide association and epistasis studies unravel the genetic architecture of sudden death syndrome resistance in soybean. Plant J..

[17] Lee Y.-G., Jeong N., Kim J.H., Lee K., Kim K.H., Pirani A., Ha B.-K., Kang S.-T., Park B.-S., Moon J.-K. (2015). Development, validation and genetic analysis of a large soybean SNP genotyping array. Plant J..

[18] Wang J., Chu S., Zhang H., Zhu Y., Cheng H., Yu D. (2016). Development and application of a novel genome-wide SNP array reveals domestication history in soybean. Sci. Rep..

[19] Saleem A., Muylle H., Aper J., Ruttink T., Wang J., Yu D., Roldán-Ruiz I. (2021). A Genome-Wide Genetic Diversity Scan Reveals Multiple Signatures of Selection in a European Soybean Collection Compared to Chinese Collections of Wild and Cultivated Soybean Accessions. Front. Plant Sci..

[20] Jeong S.-C., Moon J.-K., Park S.-K., Kim M.-S., Lee K., Lee S.R., Jeong N., Choi M.S., Kim N., Kang S.-T. (2019). Genetic diversity patterns and domestication origin of soybean. Theor. Appl. Genet..

[21] Jeong N., Kim K.-S., Jeong S., Kim J.-Y., Park S.-K., Lee J.S., Jeong S.-C., Kang S.-T., Ha B.-K., Kim D.-Y. (2019). Korean soybean core collection: Genotypic and phenotypic diversity population structure and genome-wide association study. PLoS ONE.

[22] Poland J., Endelman J., Dawson J., Rutkoski J., Wu S., Manes Y., Dreisigacker S., Crossa J., Sánchez-Villeda H., Sorrells M. (2012). Genomic Selection in Wheat Breeding using Genotyping-by-Sequencing. Plant Genome.

[23] Zhang J., Song Q., Cregan P.B., Jiang G.L. (2016). Genome-wide association study, genomic prediction and marker-assisted selection for seed weight in soybean (Glycine max). Theor. Appl. Genet..

[24] Beche E., Gillman J.D., Song Q., Nelson R., Beissinger T., Decker J., Shannon G., Scaboo A.M. (2021). Genomic prediction using training population design in interspecific soybean populations. Mol. Breed..

[25] Song Q., Yan L., Quigley C., Fickus E., Wei H., Chen L., Dong F., Araya S., Liu J., Hyten D. (2020). Soybean BARCSoySNP6K: An assay for soybean genetics and breeding research. Plant J..

[26] Contreras-Soto R.I., de Oliveira M.B., Costenaro-da-Silva D., Scapim C.A., Schuster I. (2017). Population structure, genetic relatedness and linkage disequilibrium blocks in cultivars of tropical soybean (Glycine max). Euphytica.

[27] Liu Z., Li H., Wen Z., Fan X., Li Y., Guan R., Guo Y., Wang S., Wang D., Qiu L. (2017). Comparison of Genetic Diversity between Chinese and American Soybean (Glycine max (L.)) Accessions Revealed by High-Density SNPs. Front. Plant Sci..

[28] Happ M.M., Wang H., Graef G.L., Hyten D.L. (2019). Generating High Density, Low Cost Genotype Data in Soybean [*Glycine max* (L.) Merr.]. G3 (Bethesda).

[29] Kim M.-S., Lozano R., Kim J.H., Bae D.N., Kim S.-T., Park J.-H., Choi M.S., Kim J., Ok H.-C., Park S.-K. (2021). The patterns of deleterious mutations during the domestication of soybean. Nat. Commun..

[30] Torkamaneh D., Laroche J., Valliyodan B., O’Donoughue L., Cober E., Rajcan I., Vilela Abdelnoor R., Sreedasyam A., Schmutz J., Nguyen H.T. (2021). Soybean (Glycine max) Haplotype Map (GmHapMap): A universal resource for soybean translational and functional genomics. Plant Biotechnol. J..

[31] Xu X., Bai G. (2015). Whole-genome resequencing: Changing the paradigms of SNP detection, molecular mapping and gene discovery. Mol. Breed..

[32] Huang X., Feng Q., Qian Q., Zhao Q., Wang L., Wang A., Guan J., Fan D., Weng Q., Huang T. (2009). High-throughput genotyping by whole-genome resequencing. Genome Res..

[33] Cook D.E., Lee T.G., Guo X., Melito S., Wang K., Bayless A.M., Wang J., Hughes T.J., Willis D.K., Clemente T.E. (2012). Copy Number Variation of Multiple Genes at *Rhg1* Mediates Nematode Resistance in Soybean. Science.

[34] Lam H.-M., Xu X., Liu X., Chen W., Yang G., Wong F.-L., Li M.-W., He W., Qin N., Wang B. (2010). Resequencing of 31 wild and cultivated soybean genomes identifies patterns of genetic diversity and selection. Nat. Genet..

[35] Qi X., Li M.-W., Xie M., Liu X., Ni M., Shao G., Song C., Kay-Yuen Yim A., Tao Y., Wong F.-L. (2014). Identification of a novel salt tolerance gene in wild soybean by whole-genome sequencing. Nat. Commun..

[36] Zhou Z., Jiang Y., Wang Z., Gou Z., Lyu J., Li W., Yu Y., Shu L., Zhao Y., Ma Y. (2015). Resequencing 302 wild and cultivated accessions identifies genes related to domestication and improvement in soybean. Nat. Biotechnol..

[37] Fang C., Ma Y., Yuan L., Wang Z., Yang R., Zhou Z., Liu T., Tian Z. (2016). Chloroplast DNA Underwent Independent Selection from Nuclear Genes during Soybean Domestication and Improvement. J. Genet. Genom..

[38] Maldonado dos Santos J.V., Valliyodan B., Joshi T., Khan S.M., Liu Y., Wang J., Vuong T.D., Oliveira M.F.d., Marcelino-Guimarães F.C., Xu D. (2016). Evaluation of genetic variation among Brazilian soybean cultivars through genome resequencing. BMC Genom..

[39] Fang C., Ma Y., Wu S., Liu Z., Wang Z., Yang R., Hu G., Zhou Z., Yu H., Zhang M. (2017). Genome-wide association studies dissect the genetic networks underlying agronomical traits in soybean. Genome Biol..

[40] Yang C., Yan J., Jiang S., Li X., Min H., Wang X., Hao D. (2021). Resequencing 250 Soybean Accessions: New Insights into Genes Associated with Agronomic Traits and Genetic Networks. Genom. Proteom. Bioinform..

[41] Torkamaneh D., Laroche J., Tardivel A., O’Donoughue L., Cober E., Rajcan I., Belzile F. (2018). Comprehensive description of genomewide nucleotide and structural variation in short-season soya bean. Plant Biotechnol. J..

[42] Valliyodan B., Brown A.V., Wang J., Patil G., Liu Y., Otyama P.I., Nelson R.T., Vuong T., Song Q., Musket T.A. (2021). Genetic variation among 481 diverse soybean accessions, inferred from genomic re-sequencing. Sci. Data.

[43] Kajiya-Kanegae H., Nagasaki H., Kaga A., Hirano K., Ogiso-Tanaka E., Matsuoka M., Ishimori M., Ishimoto M., Hashiguchi M., Tanaka H. (2021). Whole-genome sequence diversity and association analysis of 198 soybean accessions in mini-core collections. DNA Res..

[44] Liu Y., Du H., Li P., Shen Y., Peng H., Liu S., Zhou G.-A., Zhang H., Liu Z., Shi M. (2020). Pan-genome of wild and cultivated soybeans. Cell.

[45] Arumuganathan K., Earle E. (1991). Nuclear DNA content of some important plant species. Plant Mol. Biol. Report..

[46] Bennett M., Leitch I. Angiosperm DNA C-values database (release 8.0, Dec. 2012). http://data.kew.org/cvalues.

[47] Song Q., Jenkins J., Jia G., Hyten D.L., Pantalone V., Jackson S.A., Schmutz J., Cregan P.B. (2016). Construction of high resolution genetic linkage maps to improve the soybean genome sequence assembly Glyma1.01. BMC Genom..

[48] Valliyodan B., Cannon S.B., Bayer P.E., Shu S., Brown A.V., Ren L., Jenkins J., Chung C.Y.-L., Chan T.-F., Daum C.G. (2019). Construction and comparison of three reference-quality genome assemblies for soybean. Plant J..

[49] Shen Y., Liu J., Geng H., Zhang J., Liu Y., Zhang H., Xing S., Du J., Ma S., Tian Z. (2018). De novo assembly of a Chinese soybean genome. Sci. China Life Sci..

[50] Shen Y., Du H., Liu Y., Ni L., Wang Z., Liang C., Tian Z. (2019). Update soybean Zhonghuang 13 genome to a golden reference. Sci. China Life Sci..

[51] Li Y.-h., Zhou G., Ma J., Jiang W., Jin L.-g., Zhang Z., Guo Y., Zhang J., Sui Y., Zheng L. (2014). De novo assembly of soybean wild relatives for pan-genome analysis of diversity and agronomic traits. Nat. Biotechnol..

[52] Xie M., Chung C.Y.-L., Li M.-W., Wong F.-L., Wang X., Liu A., Wang Z., Leung A.K.-Y., Wong T.-H., Tong S.-W. (2019). A reference-grade wild soybean genome. Nat. Commun..

[53] Liu Q., Chang S., Hartman G.L., Domier L.L. (2018). Assembly and annotation of a draft genome sequence for Glycine latifolia, a perennial wild relative of soybean. Plant J..

[54] Hartman G., Wang T., Hymowitz T. (1992). Sources of resistance to soybean rust in perennial Glycine species. Plant Dis..

[55] Hartman G., Gardner M., Hymowitz T., Naidoo G. (2000). Evaluation of perennial Glycine species for resistance to soybean fungal pathogens that cause Sclerotinia stem rot and sudden death syndrome. Crop Sci..

[56] Horlock C.M., Teakle D., Jones R. (1997). Natural infection of the native pasture legume, Glycine latifolia, by a mosaic virus in Queensland. Australas. Plant Pathol..

[57] Wen L., Yuan C., Herman T., Hartman G. (2017). Accessions of perennial Glycine species with resistance to multiple types of soybean cyst nematode (*Heterodera glycines*). Plant Dis..

[58] Kim M.-S., Lee T., Baek J., Kim J.H., Kim C., Jeong S.-C. (2021). Genome Assembly of the Popular Korean Soybean Cultivar Hwangkeum. bioRxiv.

[59] Bayer P.E., Golicz A.A., Scheben A., Batley J., Edwards D. (2020). Plant pan-genomes are the new reference. Nat. Plants.

[60] Golicz A.A., Bayer P.E., Barker G.C., Edger P.P., Kim H., Martinez P.A., Chan C.K.K., Severn-Ellis A., McCombie W.R., Parkin I.A.P. (2016). The pangenome of an agronomically important crop plant Brassica oleracea. Nat. Commun..

[61] Tettelin H., Masignani V., Cieslewicz M.J., Donati C., Medini D., Ward N.L., Angiuoli S.V., Crabtree J., Jones A.L., Durkin A.S. (2005). Genome analysis of multiple pathogenic isolates of Streptococcus agalactiae: Implications for the microbial “pan-genome”. Proc. Natl. Acad. Sci. USA.

[62] Rijzaani H., Bayer P.E., Rouard M., Doležel J., Batley J., Edwards D. (2021). The pangenome of banana highlights differences between genera and genomes. Plant Genome.

[63] Ruperao P., Thirunavukkarasu N., Gandham P., Selvanayagam S., Govindaraj M., Nebie B., Manyasa E., Gupta R., Das R.R., Odeny D.A. (2021). Sorghum Pan-Genome Explores the Functional Utility for Genomic-Assisted Breeding to Accelerate the Genetic Gain. Front. Plant Sci..

[64] Montenegro J.D., Golicz A.A., Bayer P.E., Hurgobin B., Lee H., Chan C.K.K., Visendi P., Lai K., Doležel J., Batley J. (2017). The pangenome of hexaploid bread wheat. Plant J..

[65] Hurgobin B., Golicz A.A., Bayer P.E., Chan C.K.K., Tirnaz S., Dolatabadian A., Schiessl S.V., Samans B., Montenegro J.D., Parkin I.A. (2018). Homoeologous exchange is a major cause of gene presence/absence variation in the amphidiploid *Brassica napus*. Plant Biotechnol. J..

[66] Bayer P.E., Scheben A., Golicz A.A., Yuan Y., Faure S., Lee H., Chawla H.S., Anderson R., Bancroft I., Raman H. (2021). Modelling of gene loss propensity in the pangenomes of three Brassica species suggests different mechanisms between polyploids and diploids. Plant Biotechnol. J..

[67] Varshney R.K., Roorkiwal M., Sun S., Bajaj P., Chitikineni A., Thudi M., Singh N.P., Du X., Upadhyaya H.D., Khan A.W. (2021). A chickpea genetic variation map based on the sequencing of 3366 genomes. Nature.

[68] Gao L., Gonda I., Sun H., Ma Q., Bao K., Tieman D.M., Burzynski-Chang E.A., Fish T.L., Stromberg K.A., Sacks G.L. (2019). The tomato pan-genome uncovers new genes and a rare allele regulating fruit flavor. Nat. Genet..

[69] Hübner S., Bercovich N., Todesco M., Mandel J.R., Odenheimer J., Ziegler E., Lee J.S., Baute G.J., Owens G.L., Grassa C.J. (2019). Sunflower pan-genome analysis shows that hybridization altered gene content and disease resistance. Nat. Plants.

[70] Zhao J., Bayer P.E., Ruperao P., Saxena R.K., Khan A.W., Golicz A.A., Nguyen H.T., Batley J., Edwards D., Varshney R.K. (2020). Trait associations in the pangenome of pigeon pea (Cajanus cajan). Plant Biotechnol. J..

[71] Li J., Yuan D., Wang P., Wang Q., Sun M., Liu Z., Si H., Xu Z., Ma Y., Zhang B. (2021). Cotton pan-genome retrieves the lost sequences and genes during domestication and selection. Genome Biol..

[72] Zhao Q., Feng Q., Lu H., Li Y., Wang A., Tian Q., Zhan Q., Lu Y., Zhang L., Huang T. (2018). Pan-genome analysis highlights the extent of genomic variation in cultivated and wild rice. Nat. Genet..

[73] Garrison E., Sirén J., Novak A.M., Hickey G., Eizenga J.M., Dawson E.T., Jones W., Garg S., Markello C., Lin M.F. (2018). Variation graph toolkit improves read mapping by representing genetic variation in the reference. Nat. Biotechnol..

[74] Torkamaneh D., Lemay M.-A., Belzile F. (2021). The pan-genome of the cultivated soybean (PanSoy) reveals an extraordinarily conserved gene content. Plant Biotechnol. J..

[75] Bayer P.E., Valliyodan B., Hu H., Marsh J.I., Yuan Y., Vuong T.D., Patil G., Song Q., Batley J., Varshney R.K. (2021). Sequencing the USDA core soybean collection reveals gene loss during domestication and breeding. Plant Genome.

[76] Hu Z., Sun C., Lu K.-c., Chu X., Zhao Y., Lu J., Shi J., Wei C. (2017). EUPAN enables pan-genome studies of a large number of eukaryotic genomes. Bioinformatics.

[77] Hyten D.L., Song Q., Zhu Y., Choi I.-Y., Nelson R.L., Costa J.M., Specht J.E., Shoemaker R.C., Cregan P.B. (2006). Impacts of genetic bottlenecks on soybean genome diversity. Proc. Natl. Acad. Sci. USA.

[78] Zhuang Y., Wang X., Li X., Hu J., Fan L., Landis J.B., Cannon S.B., Grimwood J., Schmutz J., Jackson S.A. (2022). Phylogenomics of the genus Glycine sheds light on polyploid evolution and life-strategy transition. Nat. Plants.

[79] Joshi T., Patil K., Fitzpatrick M.R., Franklin L.D., Yao Q., Cook J.R., Wang Z., Libault M., Brechenmacher L., Valliyodan B. (2012). Soybean Knowledge Base (SoyKB): A web resource for soybean translational genomics. BMC Genom..

[80] Brown A.V., Conners S.I., Huang W., Wilkey A.P., Grant D., Weeks N.T., Cannon S.B., Graham M.A., Nelson R.T. (2021). A new decade and new data at SoyBase, the USDA-ARS soybean genetics and genomics database. Nucleic Acids Res..

[81] Karikari B., Wang Z., Zhou Y., Yan W., Feng J., Zhao T. (2020). Identification of quantitative trait nucleotides and candidate genes for soybean seed weight by multiple models of genome-wide association study. BMC Plant Biol..

[82] Rolling W., Lake R., Dorrance A.E., McHale L.K. (2020). Genome-wide association analyses of quantitative disease resistance in diverse sets of soybean [*Glycine max* (L.) Merr.] plant introductions. PLoS ONE.

[83] Klein A., Houtin H., Rond-Coissieux C., Naudet-Huart M., Touratier M., Marget P., Burstin J. (2020). Meta-analysis of QTL reveals the genetic control of yield-related traits and seed protein content in pea. Sci. Rep..

[84] Yu J., Zhang Z., Wei J., Ling Y., Xu W., Su Z. (2014). SFGD: A comprehensive platform for mining functional information from soybean transcriptome data and its use in identifying acyl-lipid metabolism pathways. BMC Genom..

[85] Gao Y., Yang Z., Yang W., Yang Y., Gong J., Yang Q.-Y., Niu X. (2021). Plant-ImputeDB: An integrated multiple plant reference panel database for genotype imputation. Nucleic Acids Res..

[86] Alkharouf N.W., Matthews B.F. (2004). SGMD: The Soybean Genomics and Microarray Database. Nucleic Acids Res..

[87] Du J., Grant D., Tian Z., Nelson R.T., Zhu L., Shoemaker R.C., Ma J. (2010). SoyTEdb: A comprehensive database of transposable elements in the soybean genome. BMC Genom..

[88] Katayose Y., Kanamori H., Shimomura M., Ohyanagi H., Ikawa H., Minami H., Shibata M., Ito T., Kurita K., Ito K. (2012). DaizuBase, an integrated soybean genome database including BAC-based physical maps. Breed. Sci..

[89] Dai X., Zhuang Z., Boschiero C., Dong Y., Zhao P.X. (2021). LegumeIP V3: From models to crops—an integrative gene discovery platform for translational genomics in legumes. Nucleic Acids Res..

[90] Grant D., Nelson R.T., Cannon S.B., Shoemaker R.C. (2010). SoyBase, the USDA-ARS soybean genetics and genomics database. Nucleic Acids Res..

[91] Goodstein D.M., Shu S., Howson R., Neupane R., Hayes R.D., Fazo J., Mitros T., Dirks W., Hellsten U., Putnam N. (2012). Phytozome: A comparative platform for green plant genomics. Nucleic Acids Res..

[92] Ma X., Yan H., Yang J., Liu Y., Li Z., Sheng M., Cao Y., Yu X., Yi X., Xu W. (2021). PlantGSAD: A comprehensive gene set annotation database for plant species. Nucleic Acids Res..

[93] Dong Q., Schlueter S.D., Brendel V. (2004). PlantGDB, plant genome database and analysis tools. Nucleic Acids Res..

[94] Deshmukh R., Rana N., Liu Y., Zeng S., Agarwal G., Sonah H., Varshney R., Joshi T., Patil G.B., Nguyen H.T. (2021). Soybean transporter database: A comprehensive database for identification and exploration of natural variants in soybean transporter genes. Physiol. Plant..

[95] Jin J., Lu P., Xu Y., Tao J., Li Z., Wang S., Yu S., Wang C., Xie X., Gao J. (2021). PCMDB: A curated and comprehensive resource of plant cell markers. Nucleic Acids Res..

[96] Ha J., Jeon H.H., Woo D.U., Lee Y., Park H., Lee J., Kang Y.J. (2019). Soybean-VCF2Genomes: A database to identify the closest accession in soybean germplasm collection. BMC Bioinform..

[97] Zeng S., Škrabišová M., Lyu Z., Chan Y.O., Bilyeu K., Joshi T. SNPViz v2.0: A web-based tool for enhanced haplotype analysis using large scale resequencing datasets and discovery of phenotypes causative gene using allelic variations. Proceedings of the 2020 IEEE International Conference on Bioinformatics and Biomedicine (BIBM).

[98] Xu Y., Guo M., Liu X., Wang C., Liu Y. (2014). SoyFN: A knowledge database of soybean functional networks. Database.

[99] Kim E., Hwang S., Lee I. (2017). SoyNet: A database of co-functional networks for soybean Glycine max. Nucleic Acids Research.

[100] Wang J., Hossain M.S., Lyu Z., Schmutz J., Stacey G., Xu D., Joshi T. (2019). SoyCSN: Soybean context-specific network analysis and prediction based on tissue-specific transcriptome data. Plant Direct.

[101] Ruprecht C., Proost S., Hernandez-Coronado M., Ortiz-Ramirez C., Lang D., Rensing S.A., Becker J.D., Vandepoele K., Mutwil M. (2017). Phylogenomic analysis of gene co-expression networks reveals the evolution of functional modules. Plant J..

[102] Tavakolan M., Alkharouf N.W., Khan F.H., Natarajan S. (2013). SoyProDB: A database for the identification of soybean seed proteins. Bioinformation.

[103] Yang J., Liu Y., Yan H., Tian T., You Q., Zhang L., Xu W., Su Z. (2018). PlantEAR: Functional Analysis Platform for Plant EAR Motif-Containing Proteins. Front. Genet..

[104] Close T.J., Wanamaker S., Roose M.L., Lyon M., Edwards D. (2007). HarvEST. Plant Bioinformatics: Methods and Protocols.

[105] Ke T., Yu J., Dong C., Mao H., Hua W., Liu S. (2015). ocsESTdb: A database of oil crop seed EST sequences for comparative analysis and investigation of a global metabolic network and oil accumulation metabolism. BMC Plant Biol..

[106] Hisano H., Sato S., Isobe S., Sasamoto S., Wada T., Matsuno A., Fujishiro T., Yamada M., Nakayama S., Nakamura Y. (2007). Characterization of the Soybean Genome Using EST-derived Microsatellite Markers. DNA Res..

[107] Umezawa T., Sakurai T., Totoki Y., Toyoda A., Seki M., Ishiwata A., Akiyama K., Kurotani A., Yoshida T., Mochida K. (2008). Sequencing and Analysis of Approximately 40 000 Soybean cDNA Clones from a Full-Length-Enriched cDNA Library. DNA Res..

[108] Seethepalli A., Guo H., Liu X., Griffiths M., Almtarfi H., Li Z., Liu S., Zare A., Fritschi F.B., Blancaflor E.B. (2020). RhizoVision Crown: An Integrated Hardware and Software Platform for Root Crown Phenotyping. Plant Phenomics.

[109] Seethepalli A., York L. (2020). RhizoVision explorer—interactive software for generalized root image analysis designed for everyone. Zenodo.

[110] Xavier A., Hall B., Hearst A.A., Cherkauer K.A., Rainey K.M. (2017). Genetic Architecture of Phenomic-Enabled Canopy Coverage in Glycine max. Genetics.

[111] Silva L.C.C., da Matta L.B., Pereira G.R., Bueno R.D., Piovesan N.D., Cardinal A.J., God P.I.V.G., Ribeiro C., Dal-Bianco M. (2021). Association studies and QTL mapping for soybean oil content and composition. Euphytica.

[112] Bhat J.A., Yu D. (2021). High-throughput NGS-based genotyping and phenotyping: Role in genomics-assisted breeding for soybean improvement. Legume Sci..

[113] Freitas Moreira F., Rojas de Oliveira H., Lopez M.A., Abughali B.J., Gomes G., Cherkauer K.A., Brito L.F., Rainey K.M. (2021). High-Throughput Phenotyping and Random Regression Models Reveal Temporal Genetic Control of Soybean Biomass Production. Front. Plant Sci..

[114] Jaiswal P., Avraham S., Ilic K., Kellogg E.A., McCouch S., Pujar A., Reiser L., Rhee S.Y., Sachs M.M., Schaeffer M. (2005). Plant Ontology (PO): A controlled vocabulary of plant structures and growth stages. Comp. Funct. Genom..

[115] Cooper L., Meier A., Laporte M.-A., Elser J.L., Mungall C., Sinn B.T., Cavaliere D., Carbon S., Dunn N.A., Smith B. (2018). The Planteome database: An integrated resource for reference ontologies, plant genomics and phenomics. Nucleic Acids Res..

[116] Papoutsoglou E.A., Faria D., Arend D., Arnaud E., Athanasiadis I.N., Chaves I., Coppens F., Cornut G., Costa B.V., Ćwiek-Kupczyńska H. (2020). Enabling reusability of plant phenomic datasets with MIAPPE 1.1. New Phytol..

[117] Cortés A.J., Blair M.W. (2018). Genotyping by Sequencing and Genome-Environment Associations in Wild Common Bean Predict Widespread Divergent Adaptation to Drought. Front. Plant Sci..

[118] Cortés A.J., Monserrate F.A., Ramírez-Villegas J., Madriñán S., Blair M.W. (2013). Drought Tolerance in Wild Plant Populations: The Case of Common Beans (*Phaseolus vulgaris* L.). PLoS ONE.

[119] López-Hernández F., Cortés A.J. (2019). Last-Generation Genome–Environment Associations Reveal the Genetic Basis of Heat Tolerance in Common Bean (*Phaseolus vulgaris* L.). Front. Genet..

[120] Buitrago-Bitar M.A., Cortés A.J., López-Hernández F., Londoño-Caicedo J.M., Muñoz-Florez J.E., Muñoz L.C., Blair M.W. (2021). Allelic Diversity at Abiotic Stress Responsive Genes in Relationship to Ecological Drought Indices for Cultivated Tepary Bean, Phaseolus acutifolius A. Gray, and Its Wild Relatives. Genes.

[121] McClean P.E., Lavin M., Gepts P., Jackson S.A., Stacey G. (2008). Phaseolus vulgaris: A Diploid Model for Soybean. Genetics and Genomics of Soybean.

[122] Shi A., Gepts P., Song Q., Xiong H., Michaels T.E., Chen S. (2021). Genome-Wide Association Study and Genomic Prediction for Soybean Cyst Nematode Resistance in USDA Common Bean (Phaseolus vulgaris) Core Collection. Front. Plant Sci..

[123] Cortés A.J., López-Hernández F., Osorio-Rodriguez D. (2020). Predicting Thermal Adaptation by Looking Into Populations’ Genomic Past. Front. Genet..

[124] Schrider D.R., Kern A.D. (2018). Supervised Machine Learning for Population Genetics: A New Paradigm. Trends Genet..

[125] Varshney R.K., Bohra A., Roorkiwal M., Barmukh R., Cowling W.A., Chitikineni A., Lam H.-M., Hickey L.T., Croser J.S., Bayer P.E. (2021). Fast-forward breeding for a food-secure world. Trends Genet..

